# Evaluation of the performance of a qPCR-based assay for HIV-1 viral load determination

**DOI:** 10.1371/journal.pone.0315579

**Published:** 2024-12-13

**Authors:** Bin Lin, Chao Han, Jin-Hai Li, Rui Wang

**Affiliations:** 1 Division of HIV/AIDS Control and Prevention, Shandong Center for Disease Control and Prevention, Jinan, Shandong Province, China; 2 Clinical Laboratory, Zibo Center for Disease Control and Prevention, Zibo, Shandong Province, China; 3 Department of Anesthesiology, Qilu Hospital of Shandong University, Jinan, Shandong Province, China; Uniformed Services University: Uniformed Services University of the Health Sciences, UNITED STATES OF AMERICA

## Abstract

**Objective:**

According to the YY/T 1182–2010 standard of the People’s Republic of China on nucleic acid amplification test reagents (kits) for medical industry, the accuracy, precision, linear range, and analytic sensitivity of HIV-1 standardized quality control products should be assessed. The Geneway HIV-1 Nucleic Acid Detection Kit from China has been successfully registered with the National Medical Products Administration. Here, we aimed to assess for the first time its detection performance.

**Methods:**

The accuracy, precision, analytic sensitivity, and linearity of the Geneway HIV-1 nucleic acid quantification test kit were analyzed using a series of diluted standard control samples of HIV-1 negative plasma. Clinical plasma samples were collected from 163 HIV-infected patients and 38 HIV-negative patients. The detection performance of the Geneway assay was compared with that of the US FDA-approved COBAS AmpliPrep/COBAS® Taqman® HIV-1 test (Roche), version 2.0, for viral load (VL) monitoring.

**Results:**

The absolute deviation of the assay between the logarithm of the measured concentration and the logarithm of the expected concentration did not exceed ±0.5 logarithmic units. All coefficients of variation (CV%) for the assays were within 5%, indicating good precision in the detection. The linearity of quantitation was excellent (r = 0.999). Overall agreement was observed in 198 of the 201 specimens (98.51%), with a kappa value of 0.953. Bland-Altman analysis revealed an average difference of 0.030 between the two assays, with 95.95% (142/148) of the differences falling within the 95% confidence limits of agreement (−0.50, 0.56). Linear regression results demonstrated a strong linear correlation between the two assays, with a high Pearson correlation coefficient (r = 0.980) and coefficient of determination (R^2^ = 0.960, *p* < 0.001).

**Conclusions:**

The Geneway HIV-1 VL assay demonstrated excellent accuracy, precision, analytic sensitivity, and linearity. Compared to the Roche assay, the Geneway assay showed good performance for HIV-1 VL detection, supporting its use in clinical practice.

## Introduction

According to the Joint United Nations Programme on HIV/AIDS (UNAIDS), 39.0 million people worldwide were living with HIV in 2022, 1.3 million were newly infected, and 630,000 died from AIDS-related illnesses [1]. By the end of 2020, there were 1.053 million reported cases of HIV infection in China, resulting in 351,000 deaths [2]. To end the AIDS epidemic by 2030, 95-95-95 targets for 2025 were set by UNAIDS in 2021, establishing goals for diagnosis, access to sustained HIV antiretroviral therapy (ART), and viral suppression for people living with HIV [3]. Viral load (VL) quantitation of HIV-1 has primarily been utilized for the monitoring of antiviral therapy, including determination of treatment timing, assessment of treatment efficacy, and early detection of drug resistance. It can also serve as a supplementary diagnostic tool for HIV infection and blood screening [4,5]. The World Health Organization (WHO) recommends routine VL testing 6 and 12 months after the start of ART and every 12 months thereafter [6]. Monitoring plasma VL levels is essential to detect treatment failure; therefore, there is an increasing global demand for widespread access to VL measurement [7,8].

Assays used to monitor HIV-1 VL have demonstrated high sensitivity and accuracy. HIV-1 is a chronic infection that requires long-term treatment and monitoring. During this time, VL measurements may be performed in various laboratories or using different assays and analyzers within the same laboratory. Therefore, it is crucial to ensure consistent, concordant, and interchangeable results across laboratories and assays. The Geneway HIV-1 Nucleic Acid Detection Kit from China has been successfully registered with the National Medical Products Administration. To the best of our knowledge, however, there have been no studies evaluating its performance. Currently, the most widely used method for HIV RNA quantification is the US FDA-approved COBAS AmpliPrep/COBAS® Taqman® HIV-1 test, version 2.0, which is based on the same principle as the Geneway assay using real-time fluorescence quantitative PCR technology. Here, we aimed to assess the Geneway assay’s performance and compare it with COBAS AmpliPrep/COBAS® Taqman® HIV-1 test, version 2.0, for VL monitoring using clinical samples. We initially assessed the accuracy, precision, analytical sensitivity, and linearity of the Geneway kit using a dilution series of HIV-1 negative plasma standard control samples. Subsequently, we compared VL results from the Geneway and Roche COBAS assays using plasma samples from 163 HIV-infected and 38 HIV-negative patients, and we assessed the concordance between the two methods with Bland-Altman analysis across different VL groups.

## Materials and methods

### Sample collection

HIV-1 quantitative quality control materials S1 (2.3E+05 IU/mL) and S3 (2.6E+03 IU/mL) as well as HIV-1 pseudovirus (1.0E+08 IU/mL) were procured from Beijing Conchestan Biotechnology Co., Ltd. According to the "GB/T21415-2008/ISO17511:2003" standard for *in vitro* diagnostic medical devices, this reference material is traceable to the WHO reference material (NIBSC code: 97/650).

In total, 201 blood samples from HIV-infected and HIV-negative patients were collected between March and June 2023 by the Shandong Provincial Center for Disease Control and Prevention. Of the 163 plasma samples that tested positive for HIV-1 antibodies, confirmation was achieved through western blot analysis. Additionally, 38 plasma samples tested negative for HIV antibodies, as determined by ELISA screening. Plasma was obtained from samples treated with EDTA to prevent coagulation and stored at −70°C after collection. Initially, the samples were thawed, mixed, and split into two tubes, with one tube allocated for COBAS testing and the other for Geneway testing. The research involving human samples complied with all relevant national regulations, institutional policies, and the tenets of the Declaration of Helsinki (as revised in 2013) and was approved by the Shandong Center for Disease Control and Prevention, Jinan, China (IRB No: SDJK-2022-048-01). The data were accessed on July 3, 2023, after obtaining ethical approval for research. Written informed consent was waived as no epidemiological investigation was involved.

### HIV-1 VL detection

The Geneway HIV-1 Nucleic Acid Quantification Detection System includes the HIV-1 Nucleic Acid Testing Reagent Kit (PCR-Fluorescent Probe Method) (Geneway, Jinan, China), an Automatic Nucleic Acid Purification Instrument (Geneway, Jinan, China), and the QuantStudio Q5 Real-time Fluorescent Quantitative PCR Instrument (ABI, USA), which features a linear quantitation range of 50–1.0E+08 IU/mL and a minimum detection limit of 30 IU/mL, were used. Results above the 50 IU/mL standard were reported as positive. The reference assay HIV-1 Nucleic Acid Quantification System includes the Roche COBAS® AmpliPrep/COBAS® TaqMan® HIV-1 test, version 2.0 (real Time-PCR; Roche, Basel, Switzerland) assay in conjunction with the COBAS® TaqMan® 48 Analyzer for automated amplification and detection (Roche, Basel, Switzerland), which features a linear quantitation range of 34–1.67E+07 IU/mL and a minimum detection limit of 34 IU/mL. Results above the 34 IU/mL standard were reported as positive. Sample preparation, RNA extraction, purification, nucleic acid amplification, and other procedures were performed according to the manufacturer’s instructions.

The accuracy, precision, analytic sensitivity, and linearity of the Geneway HIV-1 nucleic acid quantification test kit were analyzed using a series of diluted standard control samples of HIV-1 negative plasma. Nucleic acid extraction and purification, RNA isolation, reverse transcription, and quantitative real-time polymerase chain reaction (qRT-PCR) were performed according to the manufacturer’s instructions (Geneway, Jinan, China).

### Accuracy analysis

The HIV-1 quality control material S1 was diluted to achieve concentrations of 2.3E+05, 2.3E+04, 2.3E+03, and 2.3E+02 IU/mL, followed by three-fold testing for each concentration.

### Precision analysis

HIV-1 negative sample plasma was used to dilute the HIV-1 pseudo-virus sample (1.0E+08 IU/mL) to high (1.0E+04 IU/mL) and low (1.0E+03 IU/mL) concentrations. Each experiment was repeated five times with at least four parallel samples in each experiment. The results were then subjected to intra-assay testing.

### Analysis of the linear range

The HIV-1 control material at a concentration of 1.00E+08 IU/mL was diluted to produce seven concentrations: 1.00E+07, 1.00E+06, 1.00E+05, 1.00E+04, 1.00E+03, 1.00E+02, and 5.00E+01 IU/mL. Each sample was subjected to two tests.

### Analytic sensitivity analysis

The HIV-1 control material S3, initially at a concentration of 2.6E+03 IU/mL, was diluted to 3.0E+01 IU/mL and then subjected to 25 tests.

HIV-1 detection results from plasma samples using Geneway and Roche COBAS were compared, and the performance of the Geneway HIV-1 VL detection assay was evaluated. Based on the VL test outcomes from the Roche COBAS platform, positive specimens were classified into two distinct categories: those with a VL of 1000 copies/mL or higher, and those with a VL below 1000 copies/mL. Subsequently, the concordance between the two assay reagents across varying VL thresholds was evaluated. All tests were performed by the same operator.

### Statistical analysis

The HIV-1 VL (IU/mL) detection results were transformed into logarithmic values and analyzed using statistical software, including SPSS Statistics 23.0 and GraphPad Prism 8.0 (GraphPad Software, San Diego, California USA, www.graphpad.com). According to the HIV-1 RNA WHO International Standard based on Nucleic acid Technology (NIBSC 97/656), one copy of HIV-1 RNA is equivalent to 1.7±0.1 IU [9].

Based on the standard of the People’s Republic of China on nucleic acid amplification test reagents (kits) for medical industry (National Standard of the Peoples Republic China, YY/T 1182–2010), the accuracy was considered qualified if the absolute deviation of the test results did not exceed ± 0.5 logarithmic orders of magnitude. The intra-assay precision was assessed by calculating the mean, standard deviation (SD), and coefficient of variation (CV%) for each concentration, with precision deemed acceptable if the CV% for the logarithmic detection concentrations was ≤ 5%. The detection limit was considered qualified if the detection rate exceeded 95% of the lower limit in 25 repeated detections, according to the manufacturer’s instructions (Geneway, Jinan, China). A linear range was deemed qualified if the Pearson correlation coefficient (r) was ≥ 0.98.

The Cohen’s Kappa Statistic (kappa statistic) assessed the agreement in the qualitative results between the Geneway and reference assays. A kappa value of < 0.4 indicated poor agreement, 0.4 < kappa ≤ 0.75 indicated moderate agreement, and kappa > 0.75 indicated excellent agreement. The Bland-Altman model was utilized to analyze the consistency of HIV-1 VL quantification results by evaluating the deviation between the detection results of the two assays. A linear regression equation was used to evaluate the correlation between the results of the two assays. Subsequently, the Pearson correlation coefficient (r) and coefficient of determination (R^2^) were calculated, and differences were considered statistically significant at *p* < 0.05. The raw data is presented in the S1 Raw data.

## Results

### Accuracy analysis

The accuracy of the Geneway HIV-1 Test Kit was assessed by comparing the expected and measured levels of HIV-1 quantitative quality control materials at four different concentrations, ranging from 2.30E+05 to 2.30E+02 IU/mL. The results demonstrated that the absolute deviation between the measured concentration’s logarithm and the expected concentration’s logarithm did not exceed ±0.5 logarithmic units (Table 1).

**Table 1:** Analysis of the accuracy of the Geneway HIV-1 test kit.

Expected (IU/mL)	Expected [Log_10_(IU/mL)]	Mean [Log_10_(IU/mL)]	ΔLog_10_(IU/mL)
2.30E+05	5.36	5.41	0.05
2.30E+04	4.36	4.42	0.06
2.30E+03	3.36	3.40	0.04
2.30E+02	2.36	2.34	-0.02

### Precision analysis

The results showed that the intra-assay coefficient of variation was < 5% (Table 2). The low concentration level (1.0E+03 IU/mL) had a mean log_10_(IU/mL) of 3.01, with a standard deviation of 0.059 and a coefficient of variation of 2.03%. At the high concentration level (1.0E+04 IU/mL), the mean log_10_(IU/mL) was 4.00, with a standard deviation of 0.038 and a coefficient of variation of 0.98%.

**Table 2:** Analysis of the precision of the Geneway HIV-1 test kit.

	Intra-assay precision		
	Mean [Log_10_(IU/mL)]	SD [Log_10_(IU/mL)]	CV%
**Low Concentration**	3.01	0.059	2.03%
**High Concentration**	4.00	0.038	0.98%

### Analysis of the linear range

**Fig 1:**
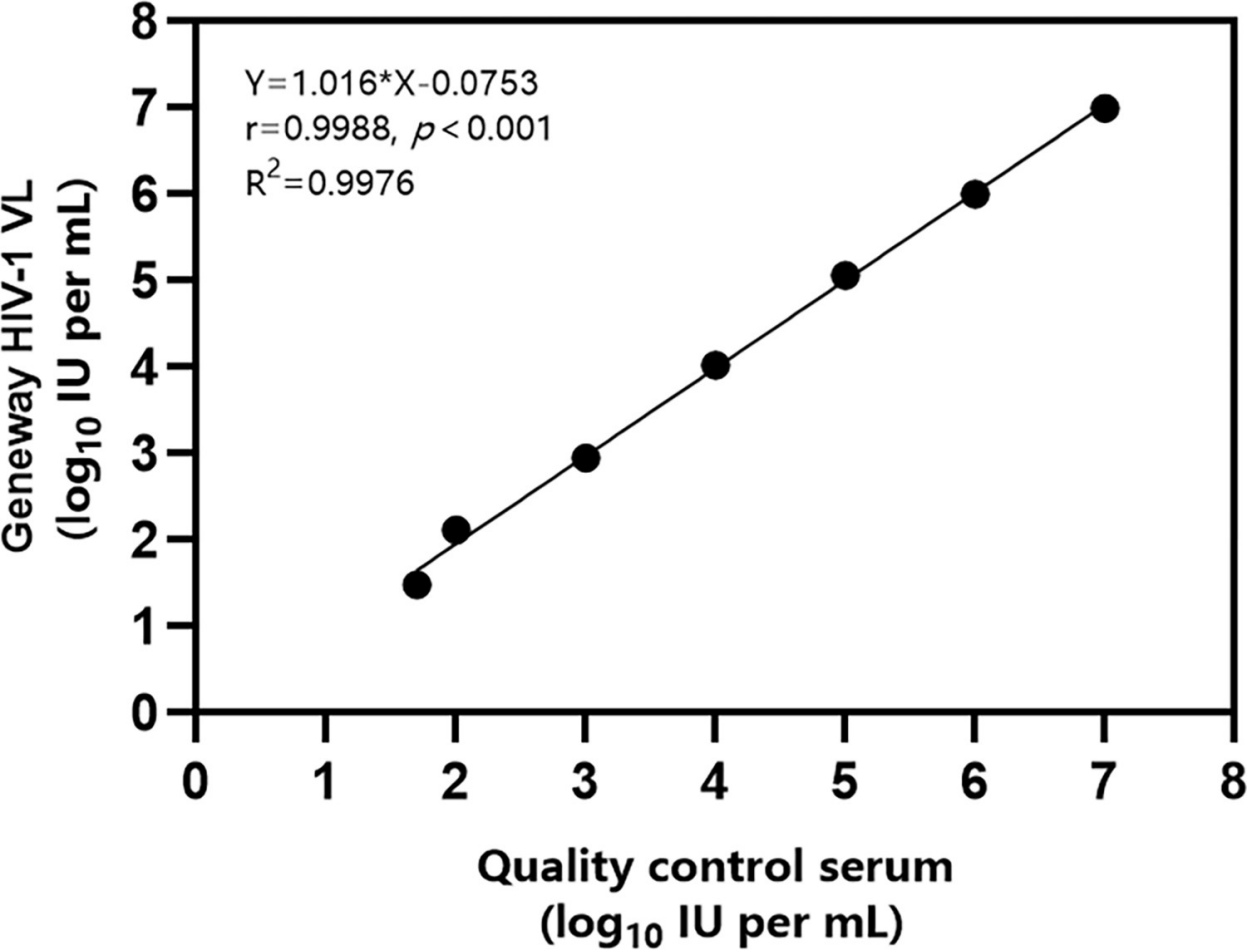
Linear relationship between the Geneway HIV-1 test results and the dilution factor.

The results indicated that the assay exhibited linearity across the concentration range of 5.00E+01 to 1.00E+07 IU/mL, with a linear slope of 1.016 and a correlation coefficient (r = 0.999) > 0.98 (Fig 1).

### Analytic sensitivity analysis

The HIV-1 control material S3, initially at a concentration of 2.6E+03 IU/mL, was diluted to 3.0E+01 IU/mL and then subjected to 25 tests. The results demonstrated a 95% detection proportion at a sample concentration of 3.0E+01 IU/mL, with the limit of quantitative detection set at the same level.

### Comparison of test results for HIV-1 infected patients between the two assays

Plasma samples from 163 patients infected with HIV-1 and 38 HIV-1-negative patients underwent qualitative analysis for HIV-1 using two distinct detection methods. The results showed that the 201 samples exhibited consistent test outcomes (Table 3). Compared to the reference assay, the positive agreement proportion with the test kit was 98.77%, whereas the negative agreement proportion was 97.44%, resulting in an overall agreement proportion of 98.51%. The Kappa Statistic revealed a value of 0.953 (*p* < 0.001), indicating a high level of consistency in qualitative detection between the two assays.

**Table 3:** Comparison of HIV‐1 infected patients results for the Geneway and reference test kits.

Geneway	Roche		Total
	Positive	Negative	
**Positive**	160	1	161
**Negative**	2	38	40
**Total**	162	39	201

Among the 161 HIV-1 positive plasma samples, 88 contained subtype information, with 46 samples classified as CRF01_AE, 26 as CRF07_BC, six as CRF08_BC, five as CRF55_ 01 B, and five as subtype B. In this study, both the Geneway and reference test kits successfully identified all 88 samples containing subtype information.

### Consistency in quantitative test results between the two assays

**Fig 2:**
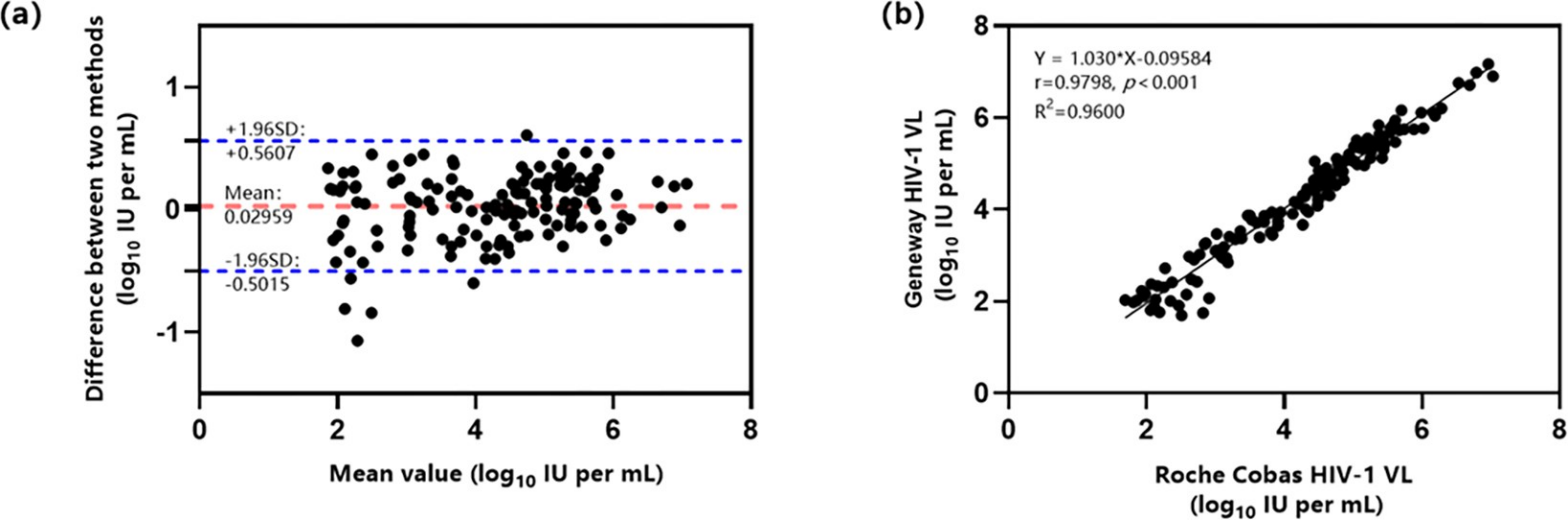
Bland‐Altman and Pearson correlation analyses of the Geneway and reference assays. (a) Bland-Altman analysis of the Geneway and reference assay test results (n = 148). The blue dashed line represents the 95% confidence interval of the difference in test results (d ± 1.96 SD, d: Mean difference, SD: Standard deviation of the difference). The red dashed line represents the average difference in test results of the two assays. (b) Linear regression analysis of the Geneway and reference test results (n = 148).

The results of 148 samples fell within the linear quantitative range of both the Geneway and Roche HIV-1 VL reagents. Bland-Altman analysis revealed an average difference of 0.030 between the two methods, with 95.95% (142/148) of the differences falling within the 95% confidence interval (−0.50, 0.56) (Fig 2A). The linear regression results demonstrated a high linear correlation between the two assays, with a Pearson correlation coefficient of r = 0.980 and a coefficient of determination of R^2^ = 0.960, *p* < 0.001 (Fig 2B).

**Fig 3:**
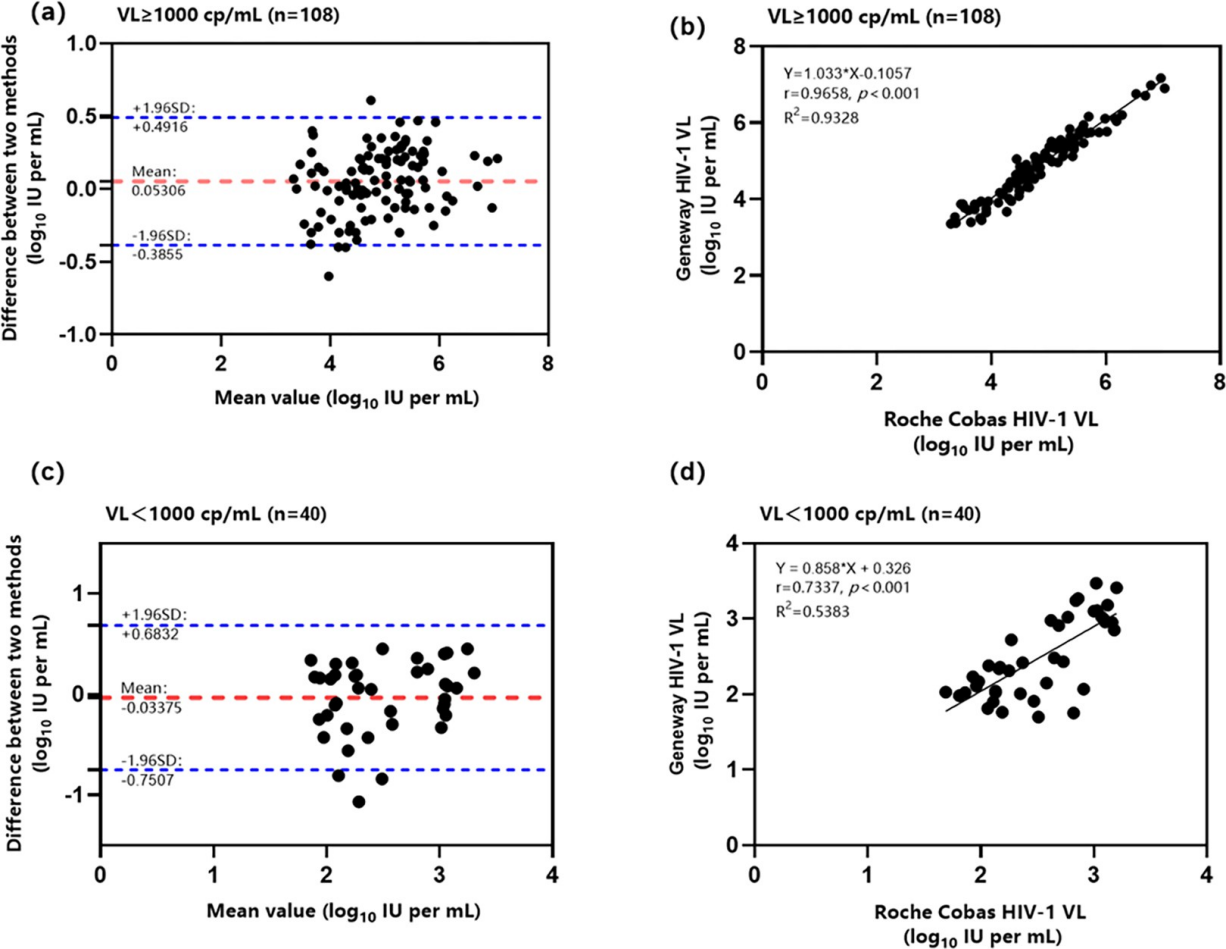
Bland‐Altman and Pearson correlation analyses for the Geneway and reference assays at different VLs. Bland-Altman (a) and linear regression (b) analyses of the Geneway and reference assay test results for the VL ≥ 1000 copies/mL group. Bland-Altman (c) and linear regression (d) analyses of the Geneway and reference assay test results for the VL< 1000 copies/mL group.

Based on the VL test results for the reference assay, 148 positive samples were stratified into VL ≥ 1000 copies/mL and VL< 1000 copies/mL groups. The Bland-Altman analysis revealed that in the VL ≥ 1000 copies/mL group, the mean difference between the two methods was 0.053, and 98.15% (106/108) of the samples exhibited differences within the 95% confidence interval (−0.39, 0.49) (Fig 3A). In the VL<1000 copies/mL group, the mean difference between the two methods was −0.034, with 92.50% (37/40) of the samples demonstrating differences within the 95% confidence interval (−0.75, 0.68; Fig 3C). The linear regression results demonstrated that in the VL ≥ 1000 copies/mL group, the two assays exhibited a strong linear correlation, with a Pearson correlation coefficient of r = 0.966, a determination coefficient of R^2^ = 0.933, and *p* < 0.001 (Fig 3B), while in the VL< 1000 copies/mL group, the two assays exhibited a Pearson correlation coefficient of r = 0.734, a determination coefficient of R^2^ = 0.538, and a *p* < 0.001 (Fig 3D).

## Discussion

VL testing plays a critical role as a laboratory test indicator for HIV prevention and control. In recent years, owing to the increasing HIV-infected population, various assays have been commonly used to measure VL in practical settings. Analyzing and comparing the test results of an assay is essential for evaluating its performance [10]. Our study evaluated the HIV-1 Geneway assay for accuracy, precision, analytic sensitivity, linearity, and detection of the common subtypes. These results demonstrated its excellent performance.

The Roche VL load assay was used as the reference assay in our study. Both assays employ real-time fluorescence PCR to monitor the PCR process by introducing a fluorescent group into the reaction system. They quantitatively detect the target genes by constructing a standard curve. HIV-1 exists throughout the world as different subtypes comprising different genetic variants [11,12]. Nevertheless, both detection methods target the gag gene and LTR region by referencing the relatively conserved sequences of the identified HIV-1 M and O groups, thereby avoiding mutation-prone regions. This substantially diminishes the likelihood of false-negative results in positive samples.

We obtained 163 plasma samples from HIV-infected patients and 38 HIV-1-negative patients, and evaluated them using two different reagents: the Geneway assay and the Roche reference assay. Qualitative test results from both assays showed good consistency. Overall agreement was observed in 198 of 201 specimens (98.51%), with a kappa value of 0.953. The Pearson’s correlation coefficient between the two assays was 0.980. The test results of the remaining three samples were inconsistent. Two plasma samples tested negative in the Geneway assay, whereas the corresponding Roche assay results were 6.51E+01 IU/mL and 2.47E+02 IU/mL. A plasma sample tested negative in the Roche assay, while the corresponding result of the Geneway assay was 5.19E+01 IU/mL. Variations in the results may be due to the selection of diverse probe sequences, reaction systems, and primers utilized in distinct assays [13]. For example, the Geneway assay requires at least 200 μL of sample, while the Roche assay requires at least 850 μL of sample. Moreover, as HIV is a retrovirus, its genomic mutation proportion is exceptionally high. Hence, mutations in the target genes of the tested sample genomes may have led to disparities between the results as well.

Automated real-time PCR technologies with an expanded dynamic range and reduced susceptibility to contamination have supplanted endpoint-based methods for VL measurement. With the decrease in the lower limits of detection and quantification of these platforms, transient low-level viremia (viral blips of 50–500 copies/mL) and persistent low-level viremia have increasingly been observed in patients undergoing ART [14–16]. Of the 40 samples with a viral load below 1,000 copies/mL analyzed in this study, 24 exhibited a viral load below 500 copies/mL. The clinical significance of low-level viremia remains a matter of debate [14,17]; however, some studies have suggested its role in predicting treatment failure and early onset of drug resistance [18]. Hence, standardizing and developing assays with a reliable readout close to the lower limit of quantification and establishing thresholds that define treatment failure are crucial for clinical decision-making.

Using the Roche assay with compatible equipment and consumables yields stable and reliable results. However, this comes with high equipment and consumable costs, a rigorous laboratory environment, and space demands. Nonetheless, its fully automated operational process is relatively straightforward and user-friendly, making it better suited for VL testing in larger medical facilities with substantial sample sizes. The Geneway assay offers a cost advantage and is compatible with commonly used real-time fluorescence quantitative PCR instruments. By adhering to the zoning concept of PCR laboratories during instrument installation, the application of domestic assays in community-level laboratories is expected to expand.

The limitation of this study is its relatively small sample size, especially for the group with VL<1000 copies/mL. Additionally, we only compared the results for two reagents and did not categorize and compare the HIV-1-related subtypes that may be involved in the test samples.

In summary, the Geneway assay assessed in this study exhibited excellent precision and a broad linear detection range, displaying a strong consistency with imported assays. Compared to imported assays, the Geneway assay offers advantages such as reduced sample input, accessible detection equipment, and affordability, enhancing its acceptability by clinical physicians and patients. However, the Geneway assay must improve the detection precision in the low VL concentration range to achieve better detection performance.

## Supporting information

S1 Raw data Supplemental data. (XLSX)

## References

[1] UNAIDS. Global HIV & AIDS statistics–Fact sheet; 2023-04-20. Available from: https://www.unaids.org/en/resources/fact-sheet.

[2] He N. Research progress in the epidemiology of HIV/AIDS in China. China CDC Wkly. 2021;3: 1022–1030. doi: 10.46234/ccdcw2021.249, , PubMed Central PMCID: PMC8633551. 34888119 PMC8633551

[3] Political declaration on HIV and AIDS: ending inequalities and getting on track to end AIDS by 2030 [EB/OL]; 2021-06-09. Available from: https://undocs.org/A/RES/75/284.

[4] Havlir D, Lockman S, Ayles H, Larmarange J, Chamie G, Gaolathe T, et al. What do the Universal Test and Treat trials tell us about the path to HIV epidemic control? J Int AIDS Soc. 2020;23: e25455. doi: 10.1002/jia2.25455, , PubMed Central PMCID: PMC7038879. 32091179 PMC7038879

[5] Miller WC, Powers KA, Smith MK, Cohen MS. Community viral load as a measure for assessment of HIV treatment as prevention. Lancet Infect Dis. 2013;13: 459–464. doi: 10.1016/S1473-3099(12)70314-6, , PubMed Central PMCID: PMC4512165. 23537801 PMC4512165

[6] Günthard HF, Aberg JA, Eron JJ, Hoy JF, Telenti A, Benson CA, et al. Antiretroviral treatment of adult HIV infection: 2014 recommendations of the International Antiviral Society-USA Panel. JAMA. 2014;312: 410–425. doi: 10.1001/jama.2014.8722, . 25038359

[7] Ma Y, Dou Z, Guo W, Mao Y, Zhang F, McGoogan JM, et al. The human immunodeficiency virus care continuum in China: 1985–2015. Clin Infect Dis. 2018;66: 833–839. doi: 10.1093/cid/cix911, . 29216405

[8] Delaney KP, DiNenno EA. HIV testing strategies for health departments to end the epidemic in the U.S. Am J Prev Med. 2021;61(5 Suppl 1): S6–S15. doi: 10.1016/j.amepre.2021.06.002, , PubMed Central PMCID: PMC9552039. 34686292 PMC9552039

[9] Holmes H, Davis C, Heath A, Hewlett I, Lelie N. An international collaborative study to establish the 1st international standard for HIV-1 RNA for use in nucleic acid-based techniques. J Virol Methods. 2001;92: 141–150. doi: 10.1016/s0166-0934(00)00283-4, . 11226561

[10] Hayden RT, Sun Y, Tang L, Procop GW, Hillyard DR, Pinsky BA, et al. Progress in quantitative viral load testing: Variability and impact of the WHO quantitative international standards. J Clin Microbiol. 2017;55: 423–430. doi: 10.1128/JCM.02044-16, , PubMed Central PMCID: PMC5277511. 27852673 PMC5277511

[11] Jiang Y, Zhang L, Hou Z, Tu A, Qiao R, Dai C, et al. Prevalence of different genotypes of HIV-1 in injection drug users in China: A systematic review and meta-analysis. Curr HIV Res. 2019;17: 240–257. doi: 10.2174/1570162X17666190919115036, . 31538898

[12] Johnson CC, Fonner V, Sands A, Ford N, Obermeyer CM, Tsui S, et al. To err is human, to correct is public health: A systematic review examining poor quality testing and misdiagnosis of HIV status. J Int AIDS Soc. 2017;20(Suppl 6): 21755. doi: 10.7448/IAS.20.7.21755, , PubMed Central PMCID: PMC5625583. 28872271 PMC5625583

[13] Klarkowski D, O’Brien DP, Shanks L, Singh KP. Causes of false-positive HIV rapid diagnostic test results. Expert Rev Anti Infect Ther. 2014;12: 49–62. doi: 10.1586/14787210.2014.866516, . 24404993

[14] Crespo-Bermejo C, de Arellano ER, Lara-Aguilar V, Valle-Millares D, Gómez-Lus ML, Madrid R, et al. Persistent low-level viremia in persons living with HIV undertreatment: An unresolved status. Virulence. 2021;12: 2919–2931. doi: 10.1080/21505594.2021.2004743, , PubMed Central PMCID: PMC8654475. 34874239 PMC8654475

[15] McCluskey SM, Siedner MJ, Marconi VC. Management of virologic failure and HIV drug resistance. Infect Dis Clin North Am. 2019;33: 707–742. doi: 10.1016/j.idc.2019.05.004, , PubMed Central PMCID: PMC6688946. 31255384 PMC6688946

[16] Santoro MM, Fabeni L, Armenia D, Alteri C, Di Pinto D, Forbici F, et al. Reliability and clinical relevance of the HIV-1 drug resistance test in patients with low viremia levels. Clin Infect Dis. 2014;58: 1156–1164. doi: 10.1093/cid/ciu020, . 24429430

[17] Chen J, He Y, Zhong H, Hu F, Li Y, Zhang Y, et al. Transcriptome analysis of CD4^+^ T cells from HIV-infected individuals receiving ART with LLV revealed novel transcription factors regulating HIV-1 promoter activity. Virol Sin. 2023;38: 398–408. doi: 10.1016/j.virs.2023.03.001, , PubMed Central PMCID: PMC10311176. 36907331 PMC10311176

[18] Li Q, Chen M, Zhao H, Yu F, Yan L, Xiao J, et al. Persistent low-level viremia is an independent risk factor for virologic failure: A retrospective cohort study in China. Infect Drug Resist. 2021;14: 4529–4537. doi: 10.2147/IDR.S332924, , PubMed Central PMCID: PMC8572020. 34754201 PMC8572020

